# Editorial: Maternal Diet and Offspring Health

**DOI:** 10.3389/fnut.2022.867661

**Published:** 2022-03-21

**Authors:** Clare M. Reynolds, Mark H. Vickers

**Affiliations:** ^1^School of Public Health, Physiotherapy and Sports Science, Conway Institute, Institute of Food and Health, University College Dublin, Dublin, Ireland; ^2^Liggins Institute, University of Auckland, Auckland, New Zealand

**Keywords:** maternal diet, offspring health, DOHaD, pregnancy, nutrition

Maternal diet is recognized as a critical factor for shaping the life-long health of the next generation. It has been over three decades since the seminal work of Barker and colleagues demonstrated that a poor early start to life was associated with an increased risk for cardiovascular disease in adulthood (1). This Developmental Origins of Health and Disease (DOHaD) paradigm has expanded, and research shows that a wide range of nutrients and dietary patterns can influence offspring development (2). Significant advancements have been made in relation to the mechanisms which dictate adverse offspring health outcomes in response to maternal diet allowing for the development of novel early life strategies for the prevention of disease. However, this is still a developing topic and more insight into the biological mechanisms which underpin a healthy start to life are required to develop guidelines and strategies to prevent cardiometabolic disease early in life.

This Research Topic “Maternal Diet and Offspring Health” highlights the diversity of exposures and experimental models in the developmental programming field. This includes human cohort studies examining gestational weight gain on infant outcomes (Li et al.; Zong et al.), murine models of maternal undernutrition (Yi et al.; Zheng et al.), murine models of maternal high fat diet (HFD) intake during pregnancy (Bolam et al.; Buckels et al.; Wang et al.) and lactation (Hafner et al.) as well as the role of sweeteners [fructose and acesulfame-k (Ace-K)] during pregnancy (Bridge-Comer et al.) and agricultural reproductive studies (Luo et al.).

Identification of exposures that impact early life developmental outcomes continues to be a major DOHaD theme. Bridge-Comer et al. examined the impact of the caloric sweetener fructose and the artificial sweetener Ace-K during pregnancy and lactation on adult offspring in a mouse model. Male and female offspring had significant sex-specific differences across metabolic outcomes. Females, but not males, born to mothers who received Ace-K had reduced glucose tolerance, compared to fructose-exposed offspring. Both sexes displayed adipocyte hypertrophy when mothers were fed sweeteners and female offspring had dysregulated ovarian gene expression and estrus cycle disruption.

While many DOHaD studies focus on pre-clinical models, the role of DOHaD in relation to agriculture is often overlooked. Luo et al. demonstrated that fermented Radix puerariae residue, a traditional Chinese medicine, increased offspring weight at weaning, improved digestive efficiency and immune profiles as well as improving overall reproductive performance in pigs.

The work by Yi et al. and Zheng et al. further highlights the impact of offspring intake of a HFD as a “second hit” to exacerbate the negative effects of intrauterine undernutrition. Yi et al. reported on the effect of maternal smoking as a risk factor for fetal growth restriction and later cardiometabolic dysregulation. Offspring of pregnant mice exposed to cigarette smoke had increased adiposity and metabolic dysfunction, effects that were amplified in the setting of a postnatal HFD. Zheng et al. focused on a mouse model of maternal low-protein induced developmental programming. They examined the impact of a postnatal diet on hypothalamic modifications and showed that maternal protein restriction combined with postnatal HFD resulted in promotor region hypomethylation and increased expression of proopiomelanocortin (POMC) in the hypothalamus of male offspring. This was linked to impaired glucose function highlighting the importance of epigenetic processes in the manifestation of offspring health risks.

Although DOHaD is often associated with outcomes related to cardiometabolic disease, poor maternal nutrition during pregnancy and lactation can have a significant effect on multiple organ systems, including the musculoskeletal system. Buckels et al. summarize the impact of maternal HFD on bone microarchitecture in offspring highlighting the potential sex-specific mechanisms which are responsible for low bone mass and microarchitecture derangement. In addition, a study by Bolam et al. examines the role of maternal HFD on supraspinatus tendons of adult rat offspring. They demonstrate an increase in tendon elasticity in male but not female offspring associated with reduced gene expression of *Col1a1* (collagen type 1) and *Scx*, a transcription factor key for tendon formation. This is the first study to identify a role for maternal HFD in the biomechanical structure of offspring tendons.

The impact of the microbiome on peripheral metabolic organs has also recently been highlighted. Wang et al. show that a diet-induced maternal obesity model altered microbiome composition in mothers which was linked to fetal liver steatosis and placental structure. With increased incidence of children presenting with fatty liver disease, this study is important in deciphering the mechanisms which underlie fetal hepatic development and contribute to long-term metabolic disease. Many DOHaD studies focus on pregnancy although lactation also represents an important critical developmental window for the setting of metabolic cues in offspring. Hafner et al. examine this time period and demonstrated that a HFD during lactation resulted in fatty liver disease and insulin resistance in male but not female offspring, effects that were partially reversed with maternal metformin treatment.

Animal studies are essential for understanding the mechanisms that underly DOHaD. However, in order to be impactful these findings must be translated to a human setting. Li et al. examine the role of maternal dietary patterns and their impact of maternal gestational weight gain and offspring birth weight in the Tongji maternal and child health cohort. They show that a dietary pattern enriched in beans and vegetables is beneficial for preventing inappropriate gestational weight gain and ensuring healthy birthweights. Given variability in human populations, ensuring appropriate experimental power is essential for reliable results. Zong et al. examine a cohort of 9 million mother-infant pairs to show that pre-pregnancy BMI is an important factor for reducing the incidence of adverse birth outcomes.

This Research Topic demonstrates the diverse range of implications for maternal diet on offspring health. Novel exposures and new insights into the potential health risks have been identified in the studies presented. Further, the combination of pre-pregnancy through to lactational dietary modifications highlight the need to consider the role of each distinct timepoint as an opportunity for intervention to prevent the long-term health consequences of exposure to adverse maternal diets. Studies presented in this topic also uncover novel mechanisms which may help to design future interventions for both mothers and offspring.

## Author Contributions

CR wrote the editorial. MV edited the editorial. All authors contributed to the article and approved the submitted version.

## Conflict of Interest

The authors declare that the research was conducted in the absence of any commercial or financial relationships that could be construed as a potential conflict of interest.

## Publisher's Note

All claims expressed in this article are solely those of the authors and do not necessarily represent those of their affiliated organizations, or those of the publisher, the editors and the reviewers. Any product that may be evaluated in this article, or claim that may be made by its manufacturer, is not guaranteed or endorsed by the publisher.
